# Early Defense Mechanisms of *Brassica oleracea* in Response to Attack by *Xanthomonas campestris* pv. *campestris*

**DOI:** 10.3390/plants10122705

**Published:** 2021-12-09

**Authors:** Lu Lu, Sokrat G. Monakhos, Yong Pyo Lim, So Young Yi

**Affiliations:** 1 Molecular Genetics and Genomics Laboratory, Department of Horticulture, College of Agriculture and Life Science, Chungnam National University, Daejeon 34134, Korea; dlLL220186@126.com (L.L.); yplim@cnu.ac.kr (Y.P.L.); 2 Moscow Timiryazev Agricultural Academy, Russian State Agrarian University, Timiryazevskaya St. 49, 127550 Moscow, Russia; smonakhos@gmail.com; 3 Institute of Agricultural Science, Chungnam National University, Daejeon 34134, Korea

**Keywords:** black rot, cabbage, disease resistance, antioxidant, *Xanthomonas campestris* pv. *campestris*

## Abstract

Black rot disease, caused by *Xanthomonas campestris* pv. *campestris* (*Xcc*), results in significant yield losses in *Brassica oleracea* crops worldwide. To find black rot disease-resistant cabbage lines, we carried out pathogenicity assays using the scissor-clipping method in 94 different *B. oleracea* lines. By comparing the lesion areas, we selected a relatively resistant line, Black rot Resistance 155 (BR155), and a highly susceptible line, SC31. We compared the two cabbage lines for the *Xcc*-induced expression pattern of 13 defense-related genes. Among them, the *Xcc*-induced expression level of *PR1* and antioxidant-related genes (*SOD*, *POD*, *APX*, *Trx H*, and *CHI*) were more than two times higher in BR155 than SC31. Nitroblue tetrazolium (NBT) and diaminobenzidine tetrahydrochloride (DAB) staining analysis showed that BR155 accumulated less *Xcc*-induced reactive oxygen species (ROS) than did SC31. In addition, 2,2-diphenyl-1-picrylhydrazyl (DPPH) radical scavenging assays showed that BR155 had higher antioxidant activity than SC31. This study, focused on the defense responses of cabbage during the early biotrophic stage of infection, indicated that *Xcc*-induced ROS might play a role in black rot disease development. We suggest that non-enzymatic antioxidants are important, particularly in the early defense mechanisms of cabbage against *Xcc*.

## 1. Introduction

Cabbage (*Brassica oleracea* L.) is an economically important crop that is widely cultivated, nutritionally rich, and valued for medicinal purposes [1,2]. Black rot disease caused by *Xanthomonas campestris* pv. *campestris* (*Xcc*) is considered the most important disease in cabbage. The pathogen enters leaves through the hydathodes or wounds and the leaf margins develop typical V-shaped lesion symptoms. Subsequently, the bacteria move via the vascular system and cause premature leaf fall, stunted growth, and even plant death [3,4,5,6,7]. As a result, the market value and yields of cabbage can be reduced severely by black rot [3,8,9].

*Xcc* has been classified into eleven races, based on the interaction between *Xcc* strains and different *Brassica* cultivars. Among them, races 1 and 4 have been reported as the most frequently occurring and most damaging in *B. oleracea* crops [10,11,12,13,14]. To curb black rot disease, several management strategies have been adopted, but none of the treatments is completely effective because of the complex survival and transmission characteristics of *Xcc* [7,15,16]. Hence, black rot-resistant hybrids/variety development and cultivation have been considered an important control method but have achieved only limited success in practice [17,18,19]. Germplasm screening to find *Xcc*-resistant cabbage cultivars is crucial for resistance breeding and is carried out mainly by phenotyping plant resources. Various inoculation methods have been used to screen for *Xcc* resistance in cabbage [20,21,22,23,24]. However, it usually takes 7–30 days from inoculation to observe obvious symptoms and evaluate the plant disease index [18,25,26]. High temperature and humidity are typically used for screening; however, such conditions are not only suitable for *Xcc* colonization, but also suitable for the growth of other pathogens, which brings greater challenges to the screening of black rot disease-resistant resources. As a consequence, it would be highly beneficial to understand the resistance mechanisms in the early response to black rot in cabbage to improve the success of resistance breeding.

Three phytohormones, salicylic acid (SA), jasmonic acid (JA), and ethylene (ET) play key roles in regulating defense signaling during responses to biotic stress. For the most part, SA mediates the defense signaling pathway involved in responses to biotrophic pathogen, whereas ET/JA mediate defense affect necrotrophic pathogens [27,28,29]. *Xcc* is a hemibiotrophic bacterial pathogen that has sequential biotrophic and necrotrophic phases. *Xcc* is generally asymptomatic while it obtains nutrients from living host tissue in the biotrophic phase, but it is relatively destructive when it damages host cells and obtains nutrients in the subsequent necrotrophic phase [7,16]. In *B. napus* responses to *Xcc*, the levels of SA and JA gradually increased, and the SA level was significantly higher than the JA level [26,30,31]. Consistently, *Xcc* strongly induced the expression of the SA signaling-mediated gene *PR1* rather than *PDF1.2*, the JA signaling response gene [26].

Reactive oxygen species (ROS) play an essential role in plant defense signaling pathways [32]. There are three major forms of ROS, superoxide anion ($O_{2^{-}}$), hydrogen peroxide (H_2_O_2_), and hydroxyl radical (OH^−^). ROS are highly reactive and toxic and can lead to the oxidative damage of cells [33]. To scavenge ROS and protect tissues from destructive reactions, plants have evolved efficient antioxidant defense systems, including antioxidant enzymes (e.g., superoxide dismutase (SOD), catalase (CAT), ascorbate peroxidase (APX), glutathione reductase (GR), and guaiacol peroxidase (GPX)), as well as non-enzymatic antioxidants (e.g., ascorbic acid, glutathione (GSH), α-tocopherol, carotenoids, phenolics, and flavonoids) [34,35]. *Xcc*-resistant cabbage lines showed lower activities of antioxidant enzymes (SOD, POX, and APX) than susceptible cabbage lines [36,37]. During the *Xcc*–*B. rapa* interaction, ROS were higher in the necrotrophic stage than in the biotrophic stage, and these findings were consistent with black rot disease progression [26,30,36].

We can observe symptoms caused by *Xcc* with the naked eye at 7-day post-inoculation (dpi). At 14 dpi, we are able to distinguish the difference between resistant and susceptible lines. In this study we selected an *Xcc*-resistant line, Black rot Resistance 155 (BR155), and a susceptible line, SC31, through comparison of symptom development. We compared and analyzed the phenotypes of BR155 and SC31, focusing on the relatively early defense responses from 24 h to 72 h after *Xcc* inoculation. Interestingly, analysis of the *Xcc*-induced expression level of *PR1* and antioxidant genes showed that it was possible to differentiate between resistant and susceptible lines within 24 h of *Xcc* inoculation. Furthermore, BR155 had relatively strong antioxidant activity, and the *Xcc*-induced ROS level was lower than that of SC31. These results led us to speculate that the regulation of ROS accumulation during early *Xcc*–cabbage interaction might be essential for restricting symptom development.

## 2. Results

### 2.1. Screening of Cabbage Germplasm for Black Rot Resistance

**Figure 1:**
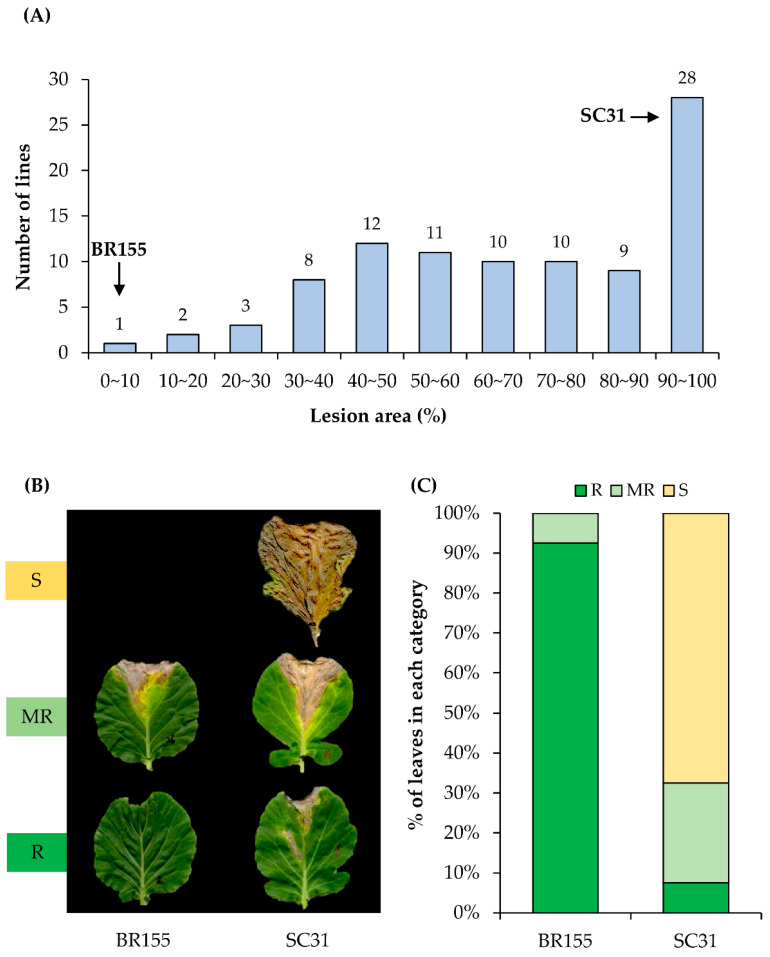
Extent of disease symptoms in 94 different *B. oleracea* cultivars 14 days after infecting with *Xcc*. (**A**) Number of lines with median lesion area percentages in each category (n ≥ 5). (**B**) Visible disease symptom development in BR155 and SC31 14 d after infecting with *Xcc*. (**C**) Stacked bar chart of the frequency percentage of three levels of black rot disease symptoms in BR155 and SC31 (n = 40). Resistant (R): leaf lesion area <20%; Moderately resistant (MR): leaf lesion area 20–40%; Susceptible (S): leaf lesion area >40%.

To identify a cabbage line harboring resistance to *Xcc*, we carried out screening of *B. oleracea* germplasm. We thought that crop wild relatives (CWRs), landraces (LRs), and heirloom lines rather than improved lines might provide a resistance source of *Xcc*. Introgression from CWRs/LRs and heirloom lines can extend the genetic base for cabbage and contribute to characteristics associated with *Xcc* resistance. A total of 94 different cabbage (*Brassica oleracea* L.) lines were tested in this study (Supplementary Table S2). Seeds were obtained from Chungnam National University (https://plus.cnu.ac.kr/, accessed on 31 March 2019), BRESOV core collection (https://bresov.eu/, accessed on 31 March 2020), and seed company (https://www.rareseeds.com/, accessed on 21 August 2016). The typical symptoms of *Xcc* on *Brassica* crops are yellow, V-shaped chlorotic lesions along the margins of leaves [3,38]. We inoculated a bacterial suspension (*Xcc* race 1) into 94 different *B. oleracea* lines using the scissor-clipping method and evaluated symptom development at 14 dpi. The size and shape of the leaves of the 94 lines of *B. oleracea* were different, so when determining the degree of disease infection, the area showing symptoms was expressed as a percentage of the total area of the inoculated leaves. As shown in Figure 1A, we observed that most of the cabbage lines used in the experiment (>60%) showed severe symptoms (30–80% of the area of the inoculated leaves). To study the defense mechanism of cabbage against *Xcc* in further detail, we selected BR155 (*Xcc*-resistant line) and SC31 (*Xcc* highly susceptible line) (Figure 1B,C).

### 2.2. Comparative Analysis of the Expression Patterns of Defense-Related Genes at the Early Infection Stage in BR155 and SC31

**Figure 2:**
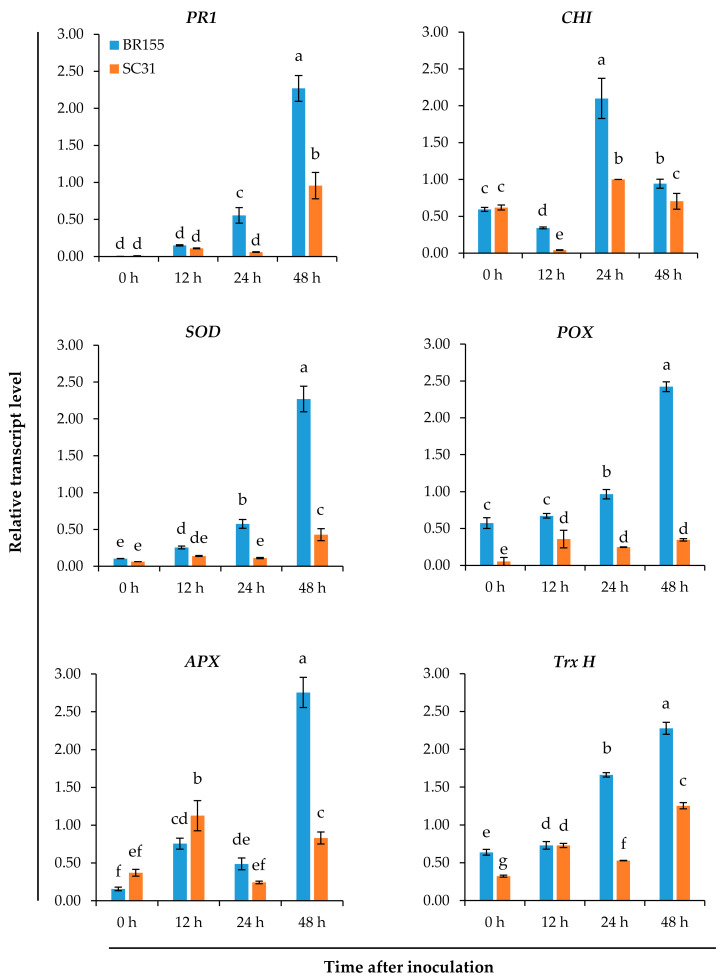
Relative transcript levels of defense-related genes (*PR1*, *CHI*, *SOD*, *POD*, *APX*, *Trx H*) in leaves of resistant (BR155) and susceptible (SC31) cabbage lines. Gene expression level was determined by qRT-PCR at the indicated time after *Xcc* inoculation and normalized to transcript levels of the 18S rRNA gene. Error bars represent standard deviations of three replicates. Similar results were obtained in at least three independent experiments. Different letters indicate significant differences among samples (α = 0.05, one-way ANOVA and Duncan’s multiple range test).

We analyzed the *Xcc*-induced expression patterns of 13 genes among the previously reported defense-related genes [37,39] using qRT-PCR, aiming to investigate the mechanism of the relatively strong resistance of BR155 to *Xcc*. The development of black rot disease showed the typical progression from biotrophic to necrotrophic stages. The initial stage of infection is biotrophic, with no visible symptoms, then the subsequent necrotrophic stage starts after at least 7 dpi and leads to V-shaped, necrotic lesions (Figure 1B). We observed the expression patterns of the *Xcc* response genes at the early infection stage (within 48 h after inoculation). Supplementary Table S1 lists the 13 *B. oleracea* genes used for qRT-PCR analysis in our study. These genes were selected from genes found to be differentially expressed in *B. rapa* and associated with resistance to *Xcc*, using suppression subtractive hybridization [37,39]. Because the previous report showed that the relative expression of each gene was enhanced in the resistant line at 12–24 h following inoculation with *Xcc* [37,39], we selected those genes to investigate the resistance of BR155 compared with SC31. As shown in Figure 2, defense-related gene expression levels, including *PR1*, were higher in BR155 (*Xcc*-resistant) than in SC31 (susceptible). Thus, we observed a difference in transcript level between BR155 and SC31 by 48 h after inoculation. Interestingly, among the genes analyzed for *Xcc*-induced expression, most of the genes strongly expressed in BR155 were antioxidant-related genes (Figure 2 and Figure S1). These data suggested that defense-related gene expression in early stage infection might influence late-stage symptom development and that antioxidants might play an important role.

### 2.3. Comparative Analysis of ROS at the Early Xcc Infection Stage in BR155 and SC31

**Figure 3:**
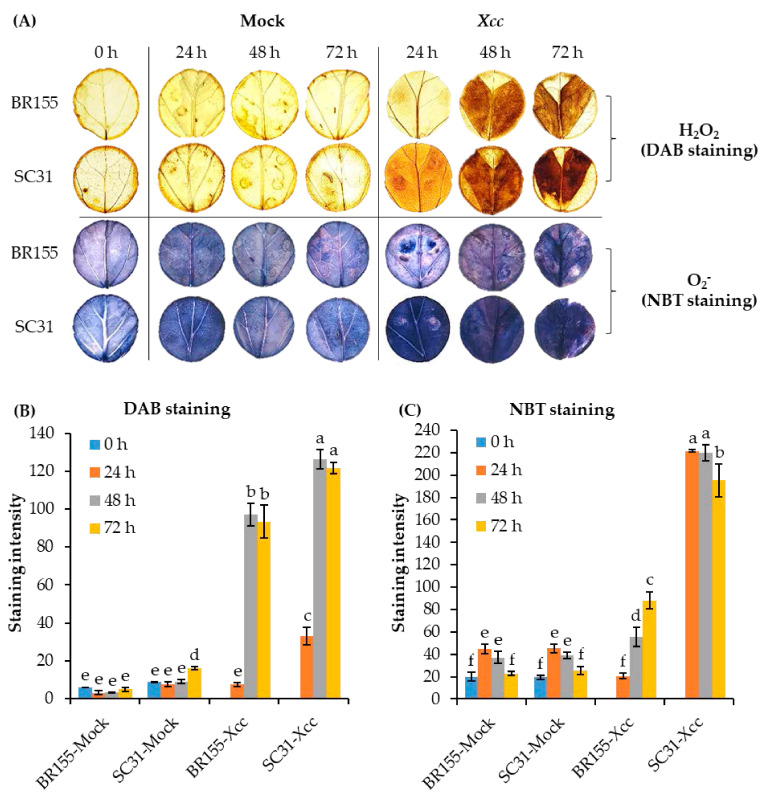
DAB and NBT staining of detached leaves of resistant (BR155) and susceptible (SC31) cabbage lines. (**A**) DAB staining for H_2_O_2_ and NBT staining for O_2_^−^ in cabbage leaves infected with *Xcc*. *Xcc* inoculum (OD_600_ = 0.01) was infiltrated into cabbage leaves. Leaf samples were harvested at 0 to 72 h after inoculation. (**B**,**C**) Staining intensity of DAB and NBT estimated using ImageJ software. Bars represent mean and error bars the standard deviation of three stained leaf discs from three different leaves. Similar results were obtained in at least three independent experiments. Different letters indicate significant differences among samples (α = 0.05, one-way ANOVA and Duncan’s multiple range test).

A high level of ROS generation leads to oxidative stress, causing a series of detrimental changes in plant cell components [40]. Plants detoxify ROS via an endogenous defensive mechanism involving antioxidant enzymes, including SOD, CAT, APX, glutathione S-transferase (GST), thioredoxins (TRXs), and others [40,41]. As shown in Figure 2, antioxidant genes (*SOD, POD, APX*, and *Trx H*) were induced by *Xcc* inoculation in both BR155 and SC31, but the expression levels of the four genes were higher in BR155 than in SC31. So then, does the resistant line BR155 produce less *Xcc*-induced ROS than the sensitive line SC31? To compare the level of *Xcc*-induced ROS between BR155 and SC31, we carried out 3-3′ diaminobenzidine (DAB) staining and nitroblue tetrazolium (NBT) staining. H_2_O_2_ can be detected with the histochemical stain DAB, which instantly forms a brownish polymer in the presence of H_2_O_2_ and peroxidase [42]. DAB staining has often been used to visualize the generation of H_2_O_2_ in plants [43]. Superoxide radical ($O_{2^{-}}$) detection and quantification were performed using NBT staining [44]. Figure 3 shows *Xcc*-induced ROS production. Positive DAB and NBT staining started after 24 h and increased to 72 h post-infection in SC31 and, as expected, SC31 presented stronger staining than did BR155. These results suggested an association between *Xcc*-induced black rot disease development and ROS production in cabbage leaves.

### 2.4. Comparative Analysis of Antioxidant Activity at the Early Xcc Infection Stage in BR155 and SC31

**Figure 4:**
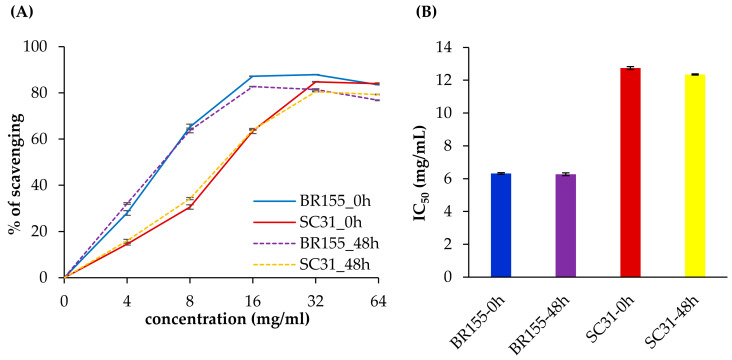
Determination of (**A**) DPPH radical-scavenging activity and (**B**) IC_50_ of methanol extracts before and 48 h after *Xcc* inoculation of cabbage lines BR155 and SC31. Error bars represent standard deviations of three replicates. Similar results were obtained in at least three independent experiments.

The *Xcc*-susceptible line, SC31, showed lower antioxidant gene expression and higher ROS accumulation than BR155 in *Xcc*-infected leaves. Therefore, we analyzed whether SC31 had lower total antioxidant activity than BR155. To study the possible role of oxidative stress in symptom development, we analyzed the ROS scavenging activities of leaf extracts of BR155 or SC31 before and 48 h after *Xcc* inoculation using free radicals of 2,2-diphenyl-1-picrylhydrazyl (DPPH) (Figure 4). In this system, standard ascorbic acid showed an IC_50_ value of 10 µg/mL (Supplementary Figure S2). The methanol extracts of both BR155 and SC31 showed a dose-dependent increase in DPPH radical-scavenging activity, as indicated by the discoloration of DPPH (Figure 4A). BR155 leaf extracts showed an IC_50_ value of 6.5 mg/mL both before and after *Xcc* inoculation. However, the SC31 leaf extract showed an IC_50_ value of 12.5–13 mg/mL, which was two-fold higher than that of BR155. Interestingly, *Xcc*-inoculated leaf extracts showed similar DPPH radical-scavenging activity to extracts before inoculation (Figure 4B). Thus, *Xcc*-infected SC31 showed relatively low total antioxidant activity, and this result might partially explain the high susceptibility of SC31 to *Xcc*.

### 2.5. Principal Component Analysis

**Figure 5:**
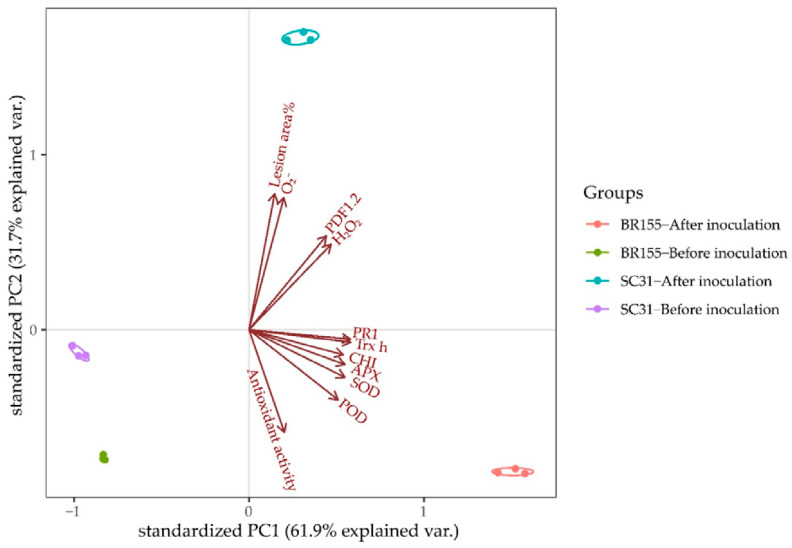
Plot of principal component analysis for *Xcc*-responsive changes in physiological and defensive parameters in resistant (BR155) and susceptible (SC31).

We performed a principal component analysis (PCA) to find the characteristics distinguishing *Xcc*-resistant and susceptible cabbage lines among physiological and defensive parameters of responses to *Xcc* infection up to 48 h after inoculation (Figure 5). The cumulative contribution of the first and the second principal components was 93.6%. Principal component 1 (PC1) explained up to 61.9% of the total variance and principal component 2 (PC2) explained 31.7% of variation. PC1 was highly contributed to by *SOD* (0.995), *POD* (0.986), *APX* (0.980), *CHI* (0.975), *Trx H* (0.961), *PR1* (0.957), and antioxidant activity (0.569).

In contrast, PC2 was highly contributed to by $O_{2^{-}}$ (0.983), lesion area % (0.977), *PDF1.2* (0.874), and H_2_O_2_ (0.835). After inoculation, apart from antioxidant activity, the other parameters of the resistant (BR155) and susceptible (SC31) lines were clustered into two groups: infected BR155 had a highly positive correlation with PC1 and was highly contributed to by antioxidant activity, namely *POD*, *SOD*, *APX*, *CHI*, *Trx H*, and *PR1*; however, it showed a negative correlation with PC2, which separated the samples on the basis of H_2_O_2_, *PDF1.2*, $O_{2^{-}}$, and lesion area %. The infected SC31 was just the opposite, and the leaf necrosis was related to the accumulation of H_2_O_2_ and $O_{2^{-}}$. These results indicated that analysis of antioxidant activity and the patterns of the antioxidant genes or induced expression of the *PR1* gene is an efficient method to identify whether cabbage lines are resistant or susceptible to *Xcc* race 1 in the early phase of infection.

## 3. Discussion

Among the races of *Xcc*, races 1 and 4 are widespread worldwide and are the most virulent, accounting for more than 90% of black rot disease [11]. However, resistance to races 1 and 4 is either non-existent or very rare in *B. oleracea* [17]. Interestingly, the interaction of *B. oleracea* and *Xcc* includes the unusual characteristic of converting from a biotrophic phase to a necrotrophic phase [26]. Necrotrophic symptoms caused by *Xcc* in cabbage leaves cannot be observed with the naked eye until at least 1 week after inoculation. Therefore, in this study, we tried to find characteristics that might distinguish resistant cabbage lines from susceptible lines among the responses during the earlier biotrophic stage of *Xcc* infection (within 3 days). First, through screening 94 *B. oleracea* accessions using the scissor-clipping method we selected cabbage line BR155, which showed strongly limited symptom development after infection by *Xcc* race 1 (Figure 1). Most cabbage lines tested were susceptible to *Xcc*, but among the 94 lines used in our study, some lines, including SC31, showed 100% lesion area in some leaves, so we categorized these lines into the highly susceptible category (Figure 1 and Supplementary Table S2).

To understand the defense mechanism of BR155, we compared the previously reported expression patterns of *Xcc*-response genes between BR155 (resistant) and SC31 (susceptible). Among the 13 genes investigated (listed in Table S1), *PR1*, several antioxidant-related genes (*SOD*, *POD*, *APX*, and *Trx H*), and *CHI* showed stronger *Xcc*-induced expression in BR155 than in SC31 (Figure 2 and Figure S1). From these results, we were able to distinguish between resistant and susceptible cabbage lines within 24 h of *Xcc* inoculation. The phytohormones SA and JA are essential for regulating plant immunity against biotic invaders [45]. Among *PR* genes, the expression of *PR1*, *PR2*, and *PR5* genes is induced by activated SA signaling, and their expression is strongly induced also after infection with biotrophic pathogens [46,47,48]. In contrast, infection by necrotrophic pathogens activated the JA-mediated defense signaling pathway and induced the expression of the *PDF1.2* gene [49]. In this study, it was *PR1* that showed higher *Xcc*-induced expression in the resistant line than *PDF1.2* 24 h after inoculation (Figure 2 and Figure S1). These results suggest that SA-mediated signaling is involved in the defense mechanism of the biotrophic stage (early infection stage) in cabbage. Amaral and colleagues compared the enzyme activity of PR proteins and antioxidant enzymes between resistant and susceptible cabbage lines from 1 to 15 d post-*Xcc* inoculation. They reported that an *Xcc*-resistant cabbage line showed relatively strong activities of PR2 and PR3 proteins but suppressed activities of antioxidant enzymes (SOD, POX, and APX) and lower concentrations of H_2_O_2_ [36]. During the interaction between *Xcc* and *B. oleracea*, we hypothesize that the defense mechanism against *Xcc* differs in the biotrophic stage (within 2 dpi) compared with the necrotrophic stage (after 7 dpi). In the previous study [36], enhanced activity of antioxidant enzymes in the *Xcc*-susceptible cabbage line compared with that of the resistant line was observed during the necrotrophic stage. Since our study focused on the defense response of *B. oleracea* in the early biotrophic stage of *Xcc* infection, the results in our study might differ from other published studies.

Biological and abiotic stresses trigger the rapid accumulation of ROS in plant tissues, and excess ROS is detrimental to many plant cell components such as lipids, proteins, and nucleic acids [33]. The plant has antioxidant systems to protect it against oxidative stress [33,50]. Among them, flavonoids can play a role in acting as UV absorbers and reducing the levels of ROS [51]. Chalcone synthase (CHS) and chalcone isomerase (CHI) are key enzymes in the flavonoid biosynthesis pathway [51,52]. We observed that the expression of the *CHI* gene increased after *Xcc* inoculation, and the *Xcc*-induced *CHI* gene expression level was higher in BR155 than in SC31, which was consistent with the expression pattern of other antioxidant genes analyzed in this study (Figure 2). ROS accumulation was observed in both BR155 and SC31 at 24 h after *Xcc* inoculation (Figure 3). However, the level of *Xcc*-induced ROS accumulation was clearly lower in BR155 than in SC31 (Figure 3). To investigate the lower ROS level in BR155 compared with SC31 after *Xcc* inoculation, we carried out a DPPH assay to evaluate non-enzymatic antioxidant activities. Yang et al. analyzed the IC_50_ value of methanol extracts of several cabbage cultivars using the DPPH assay and found values of 15 to 25 mg/mL [53]. In our system, the IC_50_ value of methanol extract of BR155 was 6.5 mg/mL, which indicated relatively high antioxidant activity. In contrast, the IC_50_ value of SC31 was 12.5–13 mg/mL, which was twice that of BR155, indicating relatively low antioxidant activity (Figure 4). Interestingly, BR155 had higher total antioxidant activity than SC31 but this difference was not related to *Xcc* infection. This result led us to speculate that the BR155 line has a higher basal level of non-enzymatic ROS scavengers than SC31. These results suggest that ROS produced by plants at the biotrophic stage (within 48 h of inoculation) might be involved in black rot disease development rather than playing a role as a plant defense signaling component. In summary, the higher *Xcc* resistance in BR155 might arise from augmented expression of antioxidant genes and strong antioxidant activity (Figure 5). The identity of the antioxidants involved in the potent antioxidant activity of BR155 is currently unknown. Therefore, in a further study to investigate the non-enzymatic antioxidants involved in the early *Xcc* resistance of BR155, we plan to analyze the levels of several ROS scavengers in BR155 and SC31.

## 4. Materials and Methods

### 4.1. Plant and Bacterial Materials

Two cabbage (*Brassica oleracea* L. var. *capitate*) cultivars were used: BR155 MS4 (a cytoplasmic male sterile line, obtained using BR155 as maintainer line via hybridization and backcrossing) and inbred line SC31. A further 20 *B. oleracea* wild relative land race (CWR-LR) lines from Korea, 23 Italian land race lines of *B. oleracea*, and 47 *B. oleracea* heirloom lines were used, which were sown and grown in plastic pots (Supplementary Table S2). *Xcc* race 1 (KACC10377) [9] was obtained from the Korean Agricultural Culture Collection.

### 4.2. Bacterial Inoculation

Bacterial inoculum was cultured on yeast extract dextrose calcium carbonate (YDC) agar medium at 30 °C for 48 h. Bacterial cells were scraped from plates and the concentration adjusted to 1.6 OD at 600 nm with 10 mM MgCl_2_ solution. The third and fourth fully expanded leaves of 35-days-old seedlings were inoculated with the bacteria by clipping and making a 4 mm incision at the leaf apex using sterile scissors dipped in the bacterial suspension. For each inoculation, the scissors were dipped into the bacterial suspension. The inoculation was performed with a completely randomized design including more than five biological replications. Fourteen days after inoculation, the black rot disease symptoms were surveyed. The following rating scales were used for visual disease estimation: Resistant (R), leaf lesion area <20%; Moderately resistant (MR), leaf lesion area 20–40%; Susceptible (S), leaf lesion area >40%. Results are expressed as: [% lesion area = (lesion area/leaf area) × 100] where the lesion V-shape is considered a triangle and the leaf a circle [54]. To determine the direct response (gene expression, ROS, and total antioxidant activity) in the infected zones, the third and fourth fully expanded leaves were inoculated by infiltrating using needleless syringes. The bacterial suspension for inoculation was adjusted to 0.01 OD at 600 nm with 10 mM MgCl_2_ solution.

### 4.3. RNA Extraction and Gene Expression Analysis by Quantitative Real-Time PCR

The infected zones of leaves were collected at 0, 12, 24, and 48 h after inoculation. For each time point, samples from five leaves were combined and considered a biological replicate. The leaves were ground to a powder in liquid nitrogen. Total RNA was extracted using the RNeasy^®^ Plant Mini Kit (Qiagen, Hilden, Germany) following the manufacturer’s instructions. The extracted RNA was cleaned using the RNeasy^®^ Plant Mini-columns (Qiagen, Hilden, Germany). The cDNAs were synthesized using 1 μg of total RNA. A real-time PCR detection system (Bio-Rad, Hercules, CA, USA) with TB Green^®^ Premix Ex Taq™ (TaKaRa, Maebashi, Japan) was used to quantify the gene expression. The sequences of gene-specific primers used for quantitative real-time PCR (qRT-PCR) are presented in Supplementary Table S1. The internal standard used was 18S rRNA. Each experiment was performed at least three times. The 2^−ΔΔCt^ method was used to quantify the relative transcript level [55].

### 4.4. ROS Analysis by DAB and NBT Staining

Histochemical detection of hydrogen peroxide (H_2_O_2_) and superoxide anion (O_2_^−^) in situ was carried out by staining with 3,3′-diaminobenzidine (DAB) tetrahydrochloride hydrate and nitroblue tetrazolium (NBT), respectively [56]. The infected leaves were harvested at 0, 24, 48, and 72 h after inoculation. Discs were punched out of the infected zones of leaves with a 2.3-cm-diameter cork borer and immersed, respectively in DAB solution (1 mg/mL, pH 3.8) and 0.1% (*w*/*v*) NBT prepared in 50 mM sodium phosphate buffer (pH 7.5). Sample tubes were wrapped in aluminum foil and the tissue infiltrated overnight at room temperature. Chlorophyll was then removed from the stained leaves with absolute ethanol and the discs vacuum-infiltrated overnight at room temperature. Finally, the leaf discs were transferred to a glass slide coated with 60% glycerol for observation. The dark brown stained patches formed by DAB reacting with endogenous H_2_O_2_ and the blue stain formed by NBT reacting with endogenous O_2_^−^ were evaluated on the depigmented discs. The staining intensity of DAB and NBT were estimated using ImageJ software (https://imagej.nih.gov/ij/download, accessed on 27 May 2021) [57]. For each time point, at least three discs originating from different plants of BR155 and SC31 were used.

### 4.5. Determination of Total Antioxidant Activity by DPPH Radical Scavenging Assay

The leaf tissues collected before inoculation (0 h) and after inoculation (48 h) were used for the preparation of plant extracts (200 mg/mL in methanol). A solution of 0.3 mM 2,2-diphenyl-1-picrylhydrazyl (DPPH) was prepared in methanol, and 680 µL of this solution was mixed with 320 µL of extract or ascorbic acid standard at different concentrations in methanol. The reaction mixture was incubated in the dark at room temperature for 30 min and the absorbance of the mixture at 517 nm was measured using a spectrophotometer. The following equation was used to calculate the percentage of DPPH radical scavenging activity:

% DPPH radical scavenging activity = [(A_0_ − A_1_)/A_0_] × 100,

where A_0_ is the absorbance of the control (containing no sample), and A_1_ is the absorbance of the extract or standard. The experiment was repeated three times at each concentration.

### 4.6. Statistical Analysis

We analyzed statistical significance by one-way analysis of variance (ANOVA) and Duncan’s multiple range test using IBM SPSS Statistics (v. 26.0, IBM, Armonk, NY, USA). Statistically significant differences at *p* < 0.05 are indicated by different letters in the figures. All physiological and defense-related parameters of the response to *Xcc*-inoculation in resistant and susceptible cabbage lines were evaluated by principal component analysis (PCA). PCA was carried out using factor analysis by IBM SPSS Statistics and the results were visualized using R script.

## 5. Conclusions

This study highlights the early responses to *Xcc* infection in resistant and susceptible cabbage lines. By analyzing the expression patterns of several *Xcc* response genes, we found that the plant antioxidant systems were involved in the early stage of cabbage defense mechanisms. Furthermore, comparative analysis of the dynamic balance of ROS in susceptible and resistant lines revealed the important role of ROS scavenging systems. These results provide new insights for research on cabbage resistance to black rot. In addition, we have shown that these early responses can be used to screen cabbage germplasm for resistance against black rot.

## Data Availability

Not applicable.

## Supplementary Materials

The following are available online at https://www.mdpi.com/article/10.3390/plants10122705/s1, Supplementary Figures: Figure S1. Relative transcript levels of defense-related marker genes in cabbage leaves at indicated times after *Xcc* inoculation. Figure S2. DPPH free radical scavenging activity of ascorbic acid (AsA). Supplementary Tables: Table S1. Primers used for gene expression analysis by qRT-PCR. Table S2. Cabbage (*Brassica oleracea* L.) germplasm for screening the resistance to *Xcc*.
