# Supplementary material for: Early Defense Mechanisms of Brassica oleracea in Response to Attack by Xanthomonas campestris pv. campestris
Source: Plants (Basel). 2021 Dec 9;10(12):2705. doi: 10.3390/plants10122705 (PMC8706934; doi:10.3390/plants10122705)
Supplement: Supplementary file 1 [file plants-10-02705-s001.zip › plants-1473582-supplementary/Supplementary Figure S1.pdf]

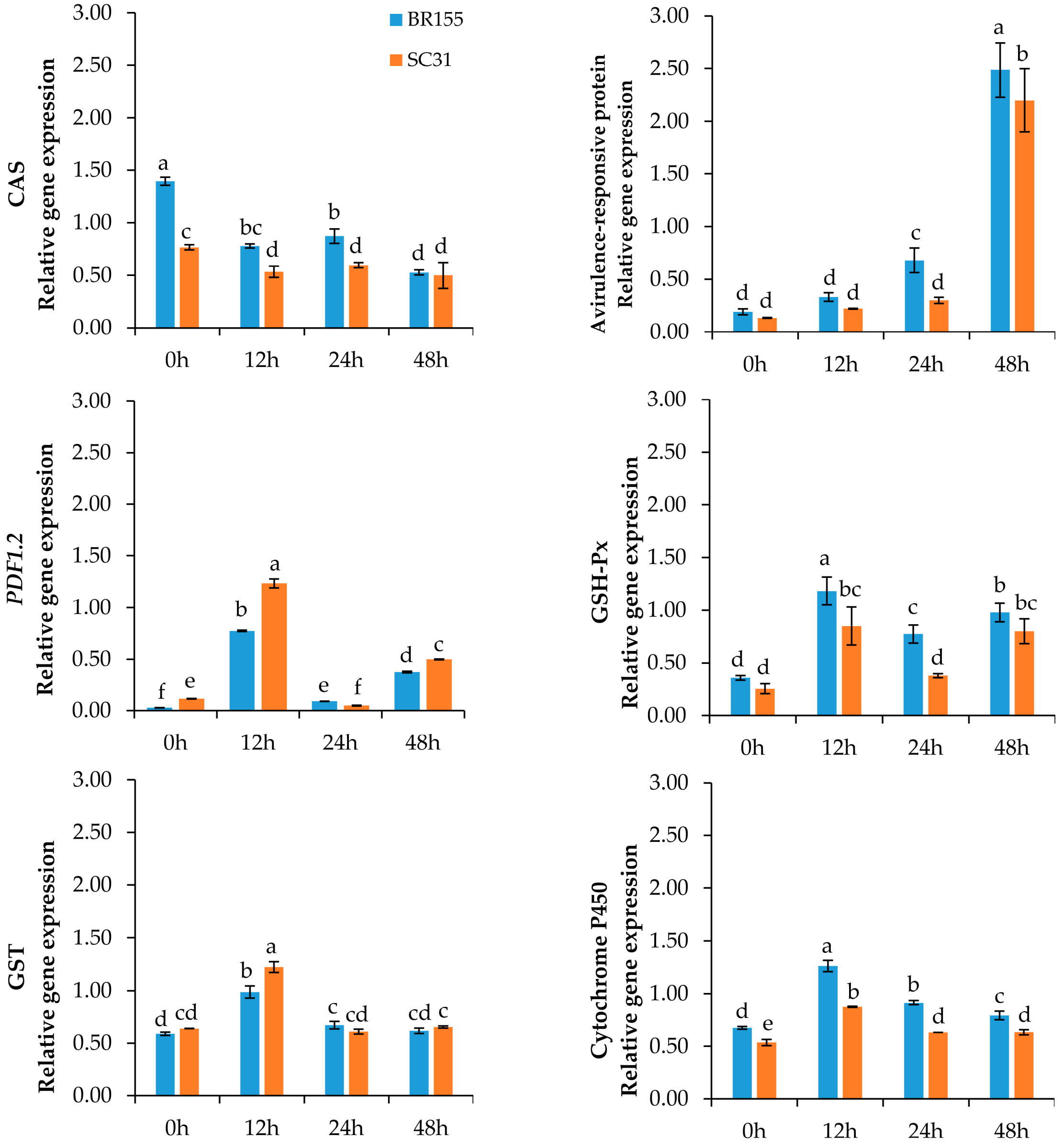

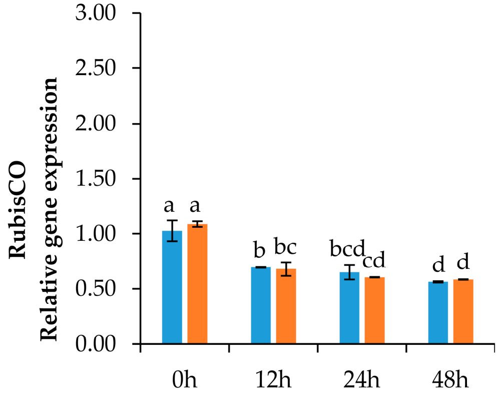

Figure S1. Relative transcripts levels of the defense-related marker genes in cabbage leaves indicated time after *Xcc* inoculation. Transcript levels of the marker genes were determined by qRT-PCR and normalized to transcript levels of 18S rRNA gene. Error bars represent standard deviations of three replicates. Similar results were obtained in at least three independent experiments. Different letters indicate significant differences among samples (α = 0.05, one-way ANOVA and Duncan's multiple range test).
