# Supplementary figures and images for: Early Defense Mechanisms of Brassica oleracea in Response to Attack by Xanthomonas campestris pv. campestris
Source: Plants (Basel). 2021 Dec 9;10(12):2705. doi: 10.3390/plants10122705 (PMC8706934; doi:10.3390/plants10122705)

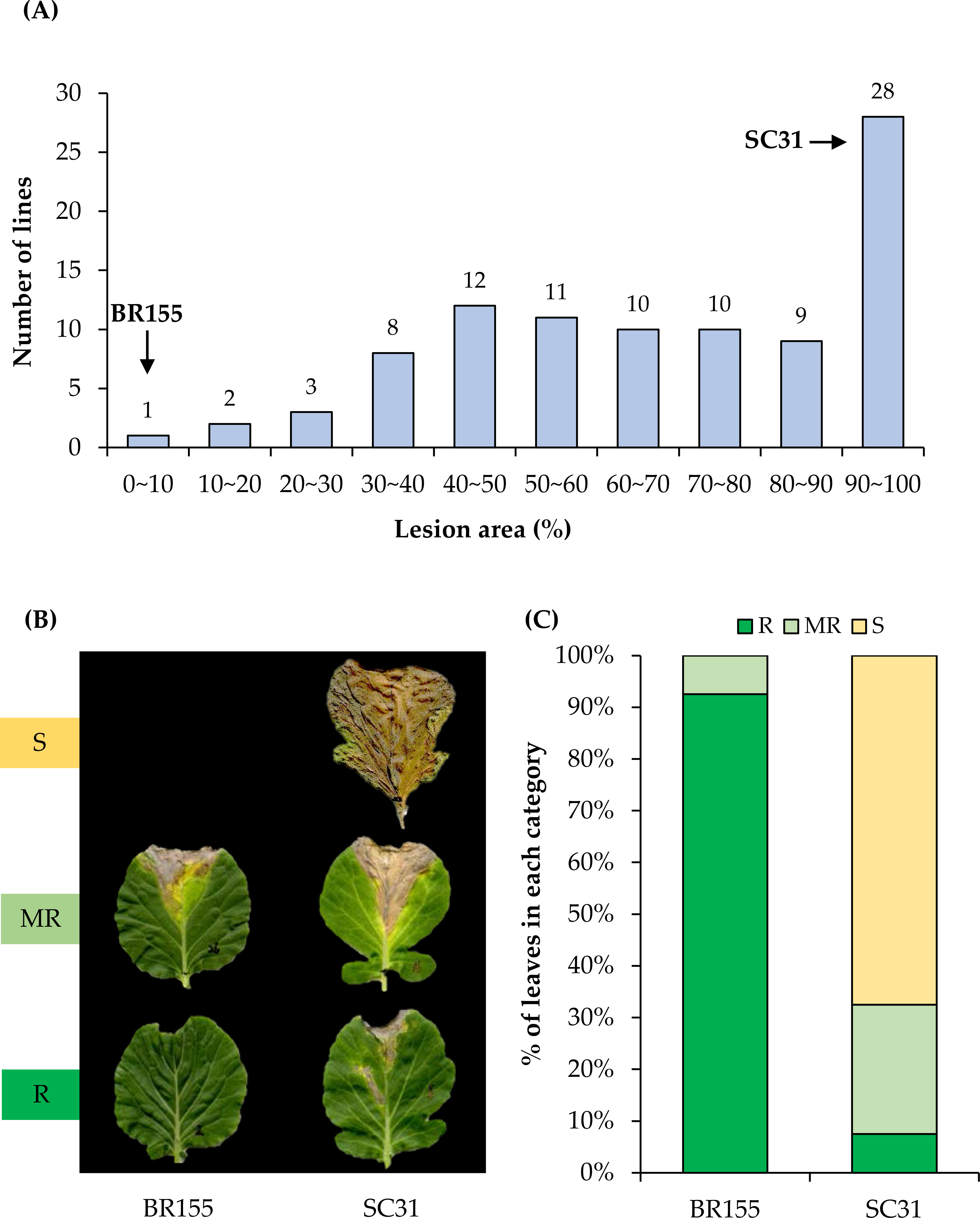

Supplement: Supplementary file 1 [file plants-10-02705-s001.zip › Figures/Figure 1.tif]

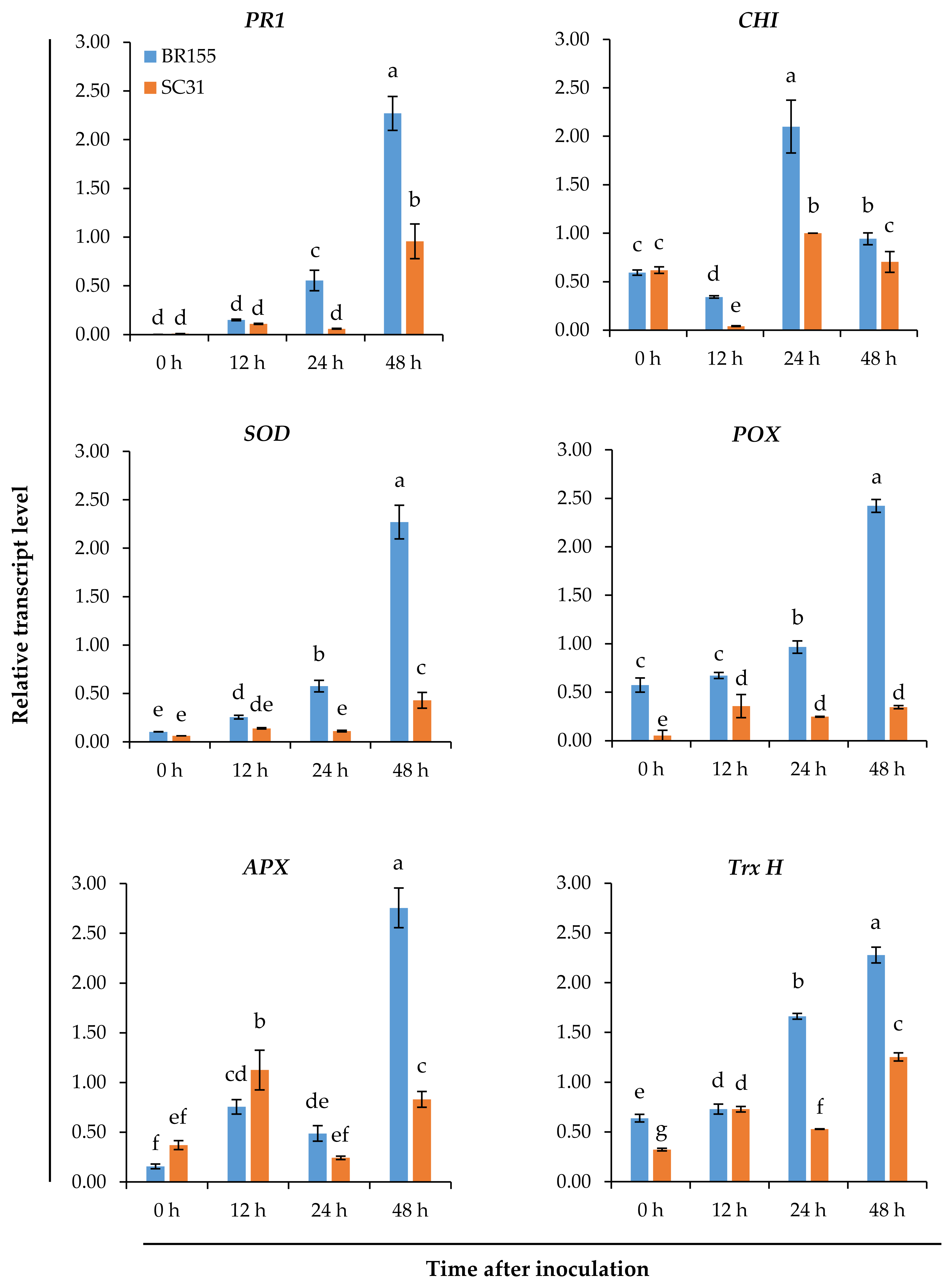

Supplement: Supplementary file 1 [file plants-10-02705-s001.zip › Figures/figure 2 revised.tif]

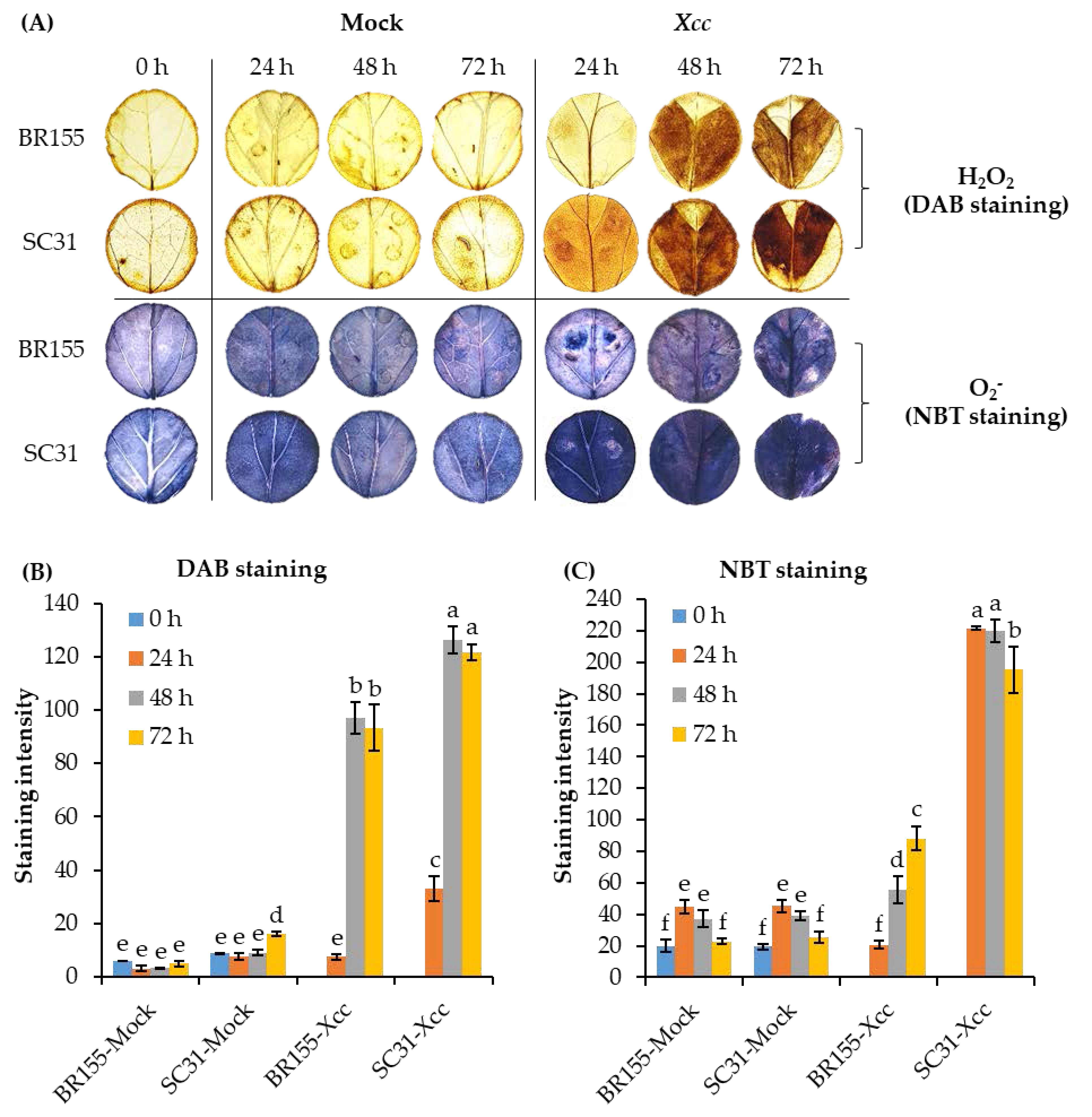

Supplement: Supplementary file 1 [file plants-10-02705-s001.zip › Figures/figure 3 revised.tif]

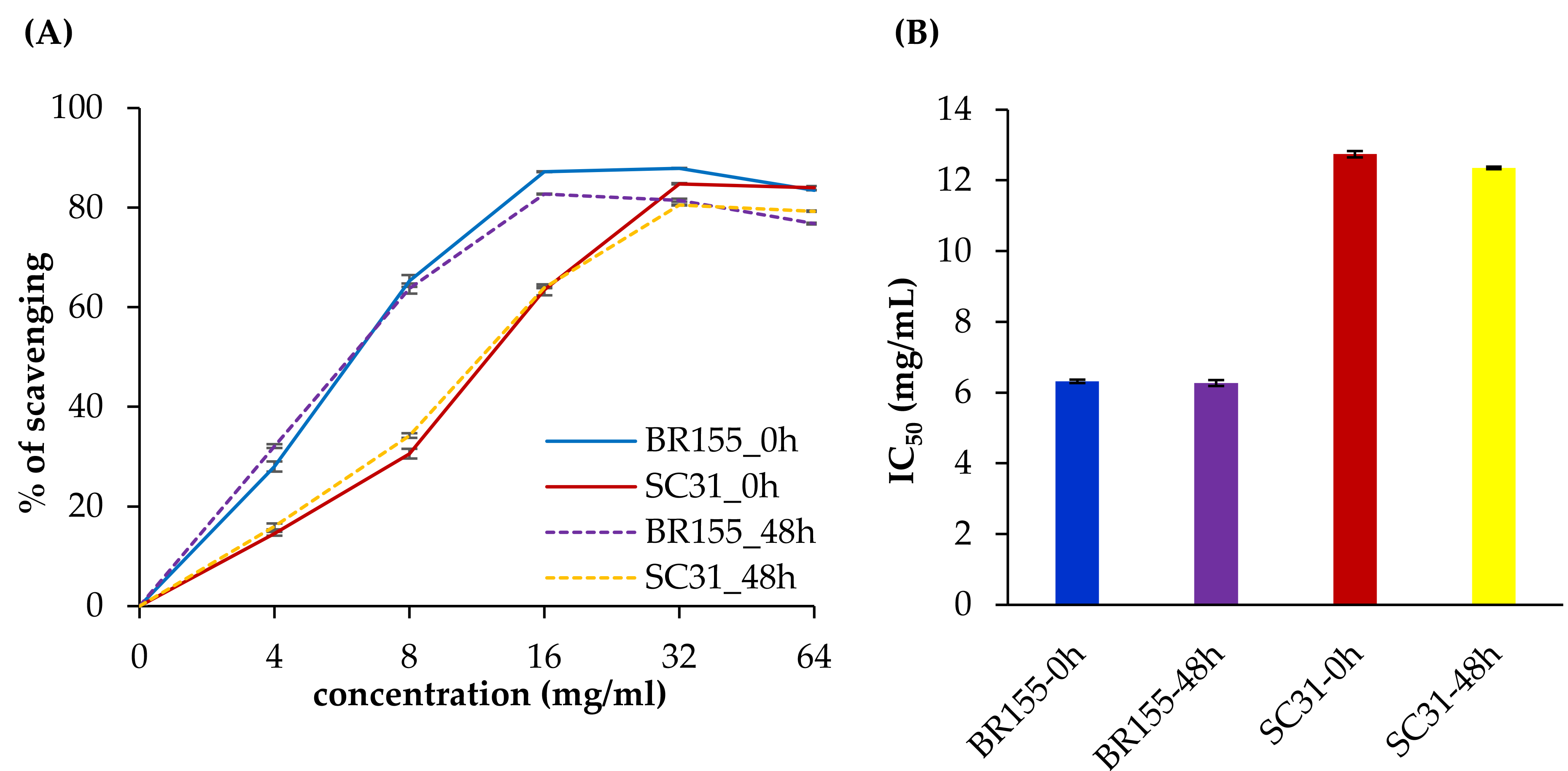

Supplement: Supplementary file 1 [file plants-10-02705-s001.zip › Figures/figure 4 revised.tif]

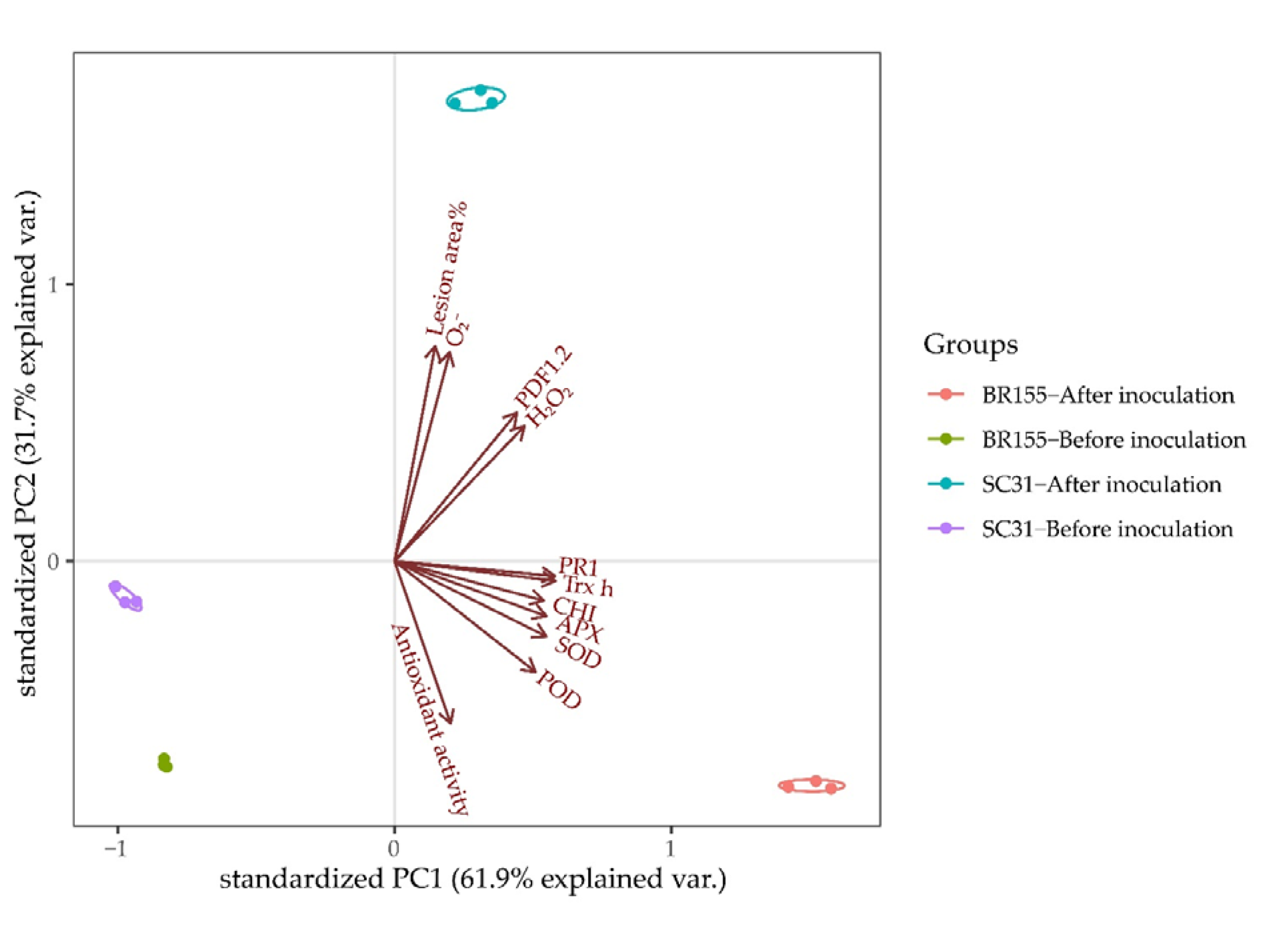

Supplement: Supplementary file 1 [file plants-10-02705-s001.zip › Figures/Figure 5.tif]

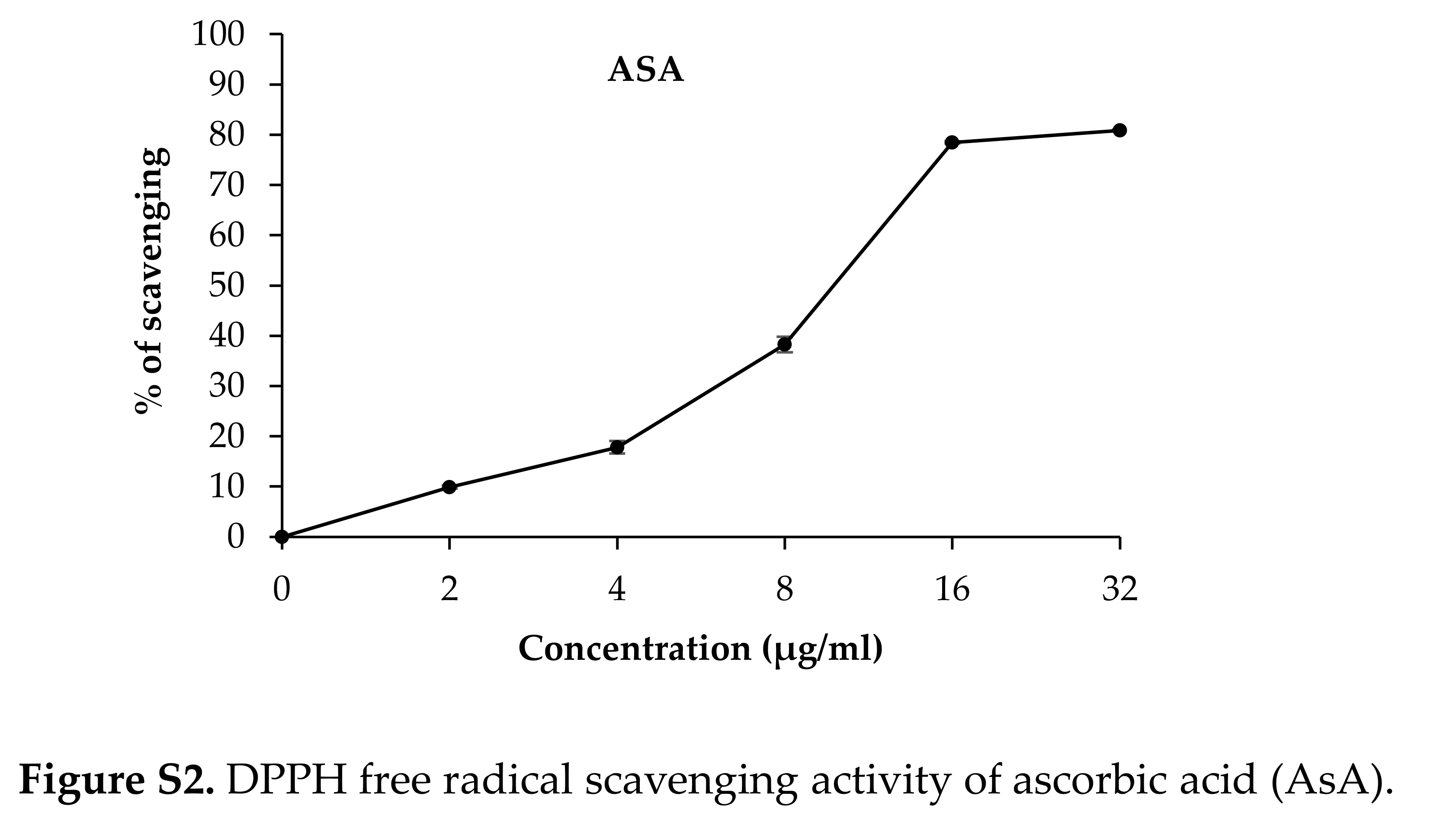

Supplement: Supplementary file 1 [file plants-10-02705-s001.zip › Figures/Supplementary Figure_2.tif]

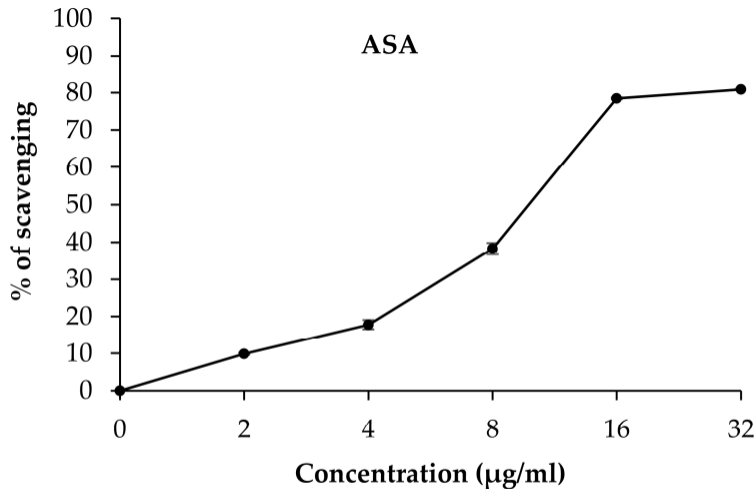

**Figure S2.** DPPH free radical scavenging activity of ascorbic acid (AsA).

Supplement: Supplementary file 1 [file plants-10-02705-s001.zip › plants-1473582-supplementary/Supplementary Figure S2.pdf]
